# Environmental Impact of Feeding with Infant Formula in Comparison with Breastfeeding

**DOI:** 10.3390/ijerph19116397

**Published:** 2022-05-24

**Authors:** Ellen Cecilie Andresen, Anne-Grete Roer Hjelkrem, Anne Kjersti Bakken, Lene Frost Andersen

**Affiliations:** 1 Department of Nutrition, University of Oslo, 0317 Oslo, Norway; l.f.andersen@medisin.uio.no; 2 Division of Food Production and Society, Norwegian Institute of Bioeconomy Research (NIBIO), 1431 Ås, Norway; anne-grete.hjelkrem@nibio.no (A.-G.R.H.); anne.kjersti.bakken@nibio.no (A.K.B.)

**Keywords:** breastfeeding, infant formula, life-cycle assessment, global warming potential, acidification, eutrophication, land use

## Abstract

Young children have unique nutritional requirements, and breastfeeding is the best option to support healthy growth and development. Concerns have been raised around the increasing use of milk-based infant formulas in replacement of breastfeeding, in regards to health, social, economic and environmental factors. However, literature on the environmental impact of infant formula feeding and breastfeeding is scarce. In this study we estimated the environmental impact of four months exclusive feeding with infant formula compared to four months exclusive breastfeeding in a Norwegian setting. We used life-cycle assessment (LCA) methodology, including the impact categories global warming potential, terrestrial acidification, marine and freshwater eutrophication, and land use. We found that the environmental impact of four months exclusive feeding with infant formula was 35–72% higher than that of four months exclusive breastfeeding, depending on the impact category. For infant formula, cow milk was the main contributor to total score for all impact categories. The environmental impact of breastfeeding was dependant on the composition of the lactating mother’s diet. In conclusion, we found that breastfeeding has a lower environmental impact than feeding with infant formula. A limitation of the study is the use of secondary LCA data for raw ingredients and processes.

## 1. Introduction

Young children have unique nutritional requirements needed to support rapid growth and development, and sufficient nutrition during these early years builds the foundation for later health [1,2]. Breastfeeding is important for infant and young child health in both high-income and low-income countries [1,2,3,4,5,6,7], and is also beneficial for the health of women [2]. In line with international recommendations, Norwegian health authorities recommend exclusive breastfeeding for 4–6 months after birth, and thereafter a gradual introduction of appropriate complementary foods with continued breastfeeding for one year or beyond [8]. Despite this, less than two out of five infants living in Norway are exclusively breastfed at four months of age, and only five percent up to six months of age [9]. Additionally, 35 percent of infants in Norway consume infant formula during the first four months of life [9].

Parents of infants and young children are facing a growing baby food market with an expanding selection of manufactured breast milk substitutes and ready-made food products. Recent studies have reported increasing global sales of milk-based formulas intended for infants and young children [10,11]. Concerns have been raised around this increasing use of milk-based formulas, both in regards to health and economic, as well as social and environmental factors [10,11,12,13,14,15,16]. Awareness of food system sustainability is high on the global political agenda [17,18]. Numerous recent reports call for more sustainable food systems and diets [19,20,21,22], including those for infants and young children [11,12].

Infant formulas are ultra-processed foods with highly complex supply-chains [11,23,24], most commonly based on combined processing of cow milk, vegetable oils, vitamins and minerals to mimic the content of human milk [25]. The ingredients are sourced from global supply chains where primary production and processing often take place in other countries than the final infant formula production [10,11,26].

Studies estimating the environmental impact of infant formula feeding and breastfeeding are limited. To our knowledge, only two previous peer-reviewed studies have compared the environmental impact of infant formula feeding and breastfeeding using life-cycle assessment (LCA) methodology [27,28]. Both studies focused solely on the impact category global warming potential, commonly known as carbon footprint. Furthermore, due to the use of different assumptions and methodologies, their conclusions are inconsistent. Karlsson et al., 2019 found that feeding with infant formula had almost twice the carbon footprint of breastfeeding [27]. Amonkar et al., 2019, on the other hand, found a higher carbon footprint of breastmilk than infant formula, assuming that breastfeeding mothers regularly expressed their breastmilk [28]. In addition, a more recent study from Pope et al., 2021 described a considerable water footprint of infant formula [12]. Other categories of environmental impact are not examined in the peer-reviewed literature on infant formula feeding and breastfeeding. Inclusion of additional impact categories may reveal potential environmental trade-offs, as the various impacts may be in conflict with one another.

The aim of the present study was to compare the environmental impact of four months exclusive feeding with infant formula with four months exclusive breastfeeding. To do this, we employed LCA methodology to evaluate the environmental impact of production and consumption of milk-based infant formula, compared with that of breastfeeding. We assessed the five environmental impact categories terrestrial acidification, marine and freshwater eutrophication, land use, and global warming potential. Sensitivity analysis of the impact of cow milk used in infant formula production is included in the assessment, as well as dietary scenarios for lactating mothers.

## 2. Materials and Methods

### 2.1. Study Set-Up

The study was undertaken in four steps. First, the environmental impact of production and distribution to retail in Norway of powdered infant formula was assessed. Secondly, the environmental impact of preparation of infant formula ready for consumption, including production and preparation of feeding bottles, was assessed. Thirdly, the environmental impact of breastfeeding was estimated. Lastly, the results were aggregated to give a total impact value for four months of feeding.

### 2.2. Life-Cycle Assessment

An LCA was performed to investigate the environmental impact of infant formula. The method assesses the environmental impact of a product or service throughout the value chain from raw material extraction to production, use, and waste treatment. The method is well-established and standardized by the International Commission for Standardization [29,30].

For impact assessment, the characterization method ReCiPe (2016) was used at midpoint level, with a hierarchist time perspective of 100 years [31]. Results for the following impact categories were included in this study: global warming potential, terrestrial acidification, freshwater eutrophication, marine eutrophication, and land use. These impact categories were included as they are systems and processes affected by food production and are commonly used in assessments of sustainable food production [19,32,33]. The impact category water use was also considered, but not included due to lack of data.

Global warming potential is a measure of how much heat a greenhouse gas traps in the atmosphere over the course of a specific time period, relative to carbon dioxide (CO_2_). It is expressed in CO_2_ equivalents (CO_2_-eq) and the gases included are methane (CH_4_), nitrous oxide (N_2_O), and some fluorinated gases, in addition to carbon dioxide. Terrestrial acidification is expressed in sulfur dioxide equivalents (SO_2_-eq) and describes changes in soil chemical properties following the deposition of nitrogen and sulfur in acidifying forms, such as nitrogen oxides, ammonia, and sulfur dioxide. Freshwater eutrophication is expressed in phosphorus equivalents (P-eq) and refers to the excessive growth of aquatic plants or algal blooms, resulting from high levels of nutrients (phosphorus and phosphate) in freshwater ecosystems. Marine eutrophication is expressed in nitrogen equivalents (N-eq) and occurs as a result of the runoff and leaching of plant nutrients from soil into freshwater and marine systems, and the subsequent rise in nutrient levels. Land use is the area of land transformed or occupied over a defined time period, and it is expressed in m^2^-eq.

The calculations of environmental impact of infant formula were carried out using the SimaPro 9.1.1.7 software (PRé Sustainability B.V., Amersfoort, The Netherlands). Production of inputs (products and energy) were included as background processes and necessary values were collected from databases. Specifically, processes with agricultural origin such as milk and vegetable oil production were sourced from the Agri-footprint 5.0 database [34,35], while all other processes were sourced from the ecoinvent 3.6 database [36,37].

The environmental impact of breastfeeding has its roots in the environmental impact of the additional food intake required for breastmilk production in lactating mothers. The data on environmental impact of food products in the mothers’ diets were taken from an LCA database published by the Dutch National Institute for Public Health and the Environment [38]. The database includes six environmental impact categories calculated with ReCiPe (2016); of these six, the present study includes the following five impact categories: global warming potential, terrestrial acidification, marine eutrophication, freshwater eutrophication, and land use.

### 2.3. Functional Units and System Boundaries

Five functional units were applied in the study. The functional units chosen for the analysis were 1 kg powdered infant formula at retail in Norway, 1 kg infant formula ready for consumption in Norway, 1 kg breastmilk from a woman in Norway, four months exclusive feeding with infant formula, and four months exclusive breastfeeding. A functional unit of 1 kg was chosen in order to facilitate comparison between the different feeding alternatives and allow for calculation of various consumption scenarios for partial or exclusive bottle feeding, i.e., consumption over a four month period.

The system boundaries chosen for the LCA of infant formula covered processes within all stages of the product life cycle spanning from cradle to grave, including raw material extraction through materials processing, manufacturing, distribution, and use.

The system boundaries applied in the database used for estimating the environmental impact of breastfeeding covered all stages of a food product’s life cycle from cradle to grave, including primary production, processing, and distribution, as well as preparation, consumption, and waste at the household level, and thus correspond with the system boundaries used in the LCA on infant formula.

### 2.4. Production of Powdered Infant Formula

#### 2.4.1. Infant Formula Recipe and Ingredients

Infant formula intended for infants 0–6 months was chosen in the present study, as this is the type of formula that competes most with breastfeeding. Infant formulas are sold as powder, liquid concentrate or liquid ready-to-drink. We assessed powder as this is commonly sold and consumed.

The majority of infant formulas are based on cow milk, which is further processed into skimmed milk, whey, and lactose, and blended with vegetable oils, minerals, and vitamins [25]. Compared to human milk, cow milk has a higher content of protein and saturated fat and a lower content of carbohydrates and unsaturated fat [25]. The cow milk is therefore skimmed and vegetable oils are added to reduce the saturated fatty acid content and increase the unsaturated fatty acid content [39]. To obtain a comparable protein and carbohydrate composition additional whey protein and lactose are added. Lastly, a mix of vitamins and minerals are added to satisfy nutritional requirements.

A baseline recipe was assumed, similar to the recipe described in Karlsson et al. [27]. The recipe is based on a literature survey, and the composition is within the ranges provided by the Joint FAO/WHO Codex Alimentarius standards of energy, protein, fat, and carbohydrates [40]. According to Codex standards, 100 mL of prepared infant formula should provide 60–70 kcal of energy; and, 100 kcal prepared infant formula should contain 9.0–14.0 g carbohydrates, 1.8–3 g proteins and 4.4–6.0 g lipids. Further, the recipe contains a composition of energy, protein, fat, and carbohydrate that is in accordance with the type of infant formula most commonly used in Norway [9,41].

The main ingredients of infant formula powder were identified to be skimmed milk, whey protein concentrate, lactose, and vegetable oils, see Table 1. Minerals and vitamins were excluded from the assessment, since they are unlikely to significantly affect environmental impact scores [27].

Unprocessed raw milk is the baseline for the infant formula ingredients skimmed milk, whey protein concentrates, and lactose. Adapted from Karlsson et al., 2019, a total of 6.6 kg energy corrected raw milk is required to produce 1 kg infant formula powder [27]. Here, a weight percentage of food losses and waste in postharvest handling and storage of 0.5% was included, as estimated for Europe in Gustavsson et al. [42]. The most commonly consumed infant formula in Norway [9] is produced in the Netherlands using raw milk from the Netherlands [43]. LCA data on raw milk production was obtained from the database Agri-footprint, and represented an average Dutch dairy farm. The emissions from meat and milk were allocated according to economic value.

Based on the estimated baseline recipe, the total amount of vegetable oils required to produce 1 kg of infant formula powder is 0.25 kg (Table 1). The vegetable oil in the baseline recipe consists of a mixture of sunflower and rapeseed oil, 85 and 15%, respectively [44]. LCA data on sunflower and rapeseed oil production was obtained from the database Agri-footprint, representing the market mix sold in the Netherlands. Economic allocation was used to divide emissions between the two products produced in oil production: oil and meal for animal feed.

#### 2.4.2. Infant Formula Processing and Packaging

**Figure 1:**
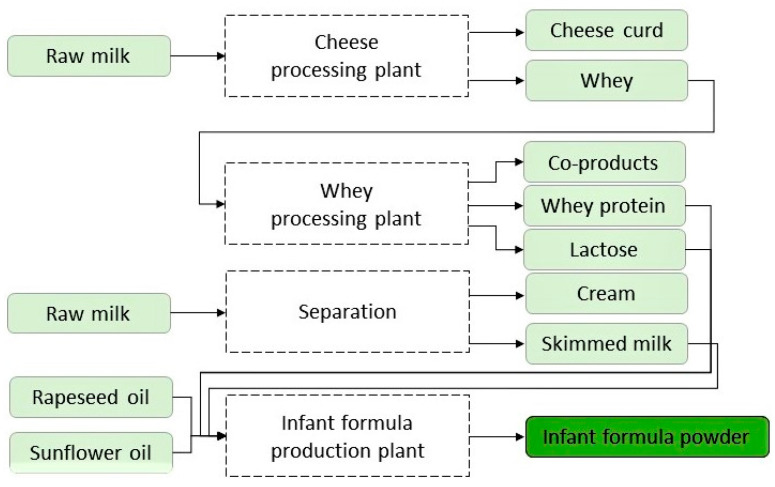
The processes involved in the production of infant formula included in the life-cycle assessment, adapted from Karlsson et al., 2019 [27]. The amount of raw ingredients (raw milk, rapeseed oil, and sunflower oil) and energy used for processing of 1 kg infant formula powder is provided in Table S1.

Data on energy requirements for the production of infant formula were taken from Karlsson et al. [27], and a rough overview of the processes are provided in Figure 1. Powdered infant formula can either be produced by a dry-blending process or a wet mixing and spray drying process. According to Karlsson et al., 2019 [27], the latter process is preferred as it is easier to ensure that the ingredients remain free of contaminants. This method was therefore represented in this study.

Production of infant formula requires several sub-processes, including production of skimmed milk, whey protein, and lactose from raw milk, before these ingredients are mixed with vegetable oil (and vitamins and minerals) at the infant formula production plant. The required total energy for production was included in the study as a sum of the values from the different production steps. National electricity mix from the Netherlands, as provided in the ecoinvent database, was used, with shares of electricity technologies valid for the year 2016. For natural gas, European averages without Switzerland were selected from the same database.

The infant formula most commonly consumed in Norway is packed in tin cans [41] and this packaging was thus included in our analysis. Each tin can holds 0.8 kg infant formula powder and 0.14 kg canning steel is required per kg infant formula powder. Data on production of the tin cans was included through production of the material, thin plated chromium steel. The LCA values were obtained from the ecoinvent database.

#### 2.4.3. Transport and Distribution

Raw milk was assumed to be transported 100 km by refrigerated road transport between farm and processing plant before production of the milk-based ingredients, skimmed milk, lactose, and whey protein. Subsequently, the ingredients were assumed to be transported 100 km by road transport to the infant formula production plant. Transport of vegetable oils was adapted directly through the market mix product used from the ecoinvent database.

We assumed that the infant formula sold in Norway is produced in the Netherlands, transported first 100 km by road from production plant to the port in Amsterdam, and finally 1000 km by sea from Amsterdam to Oslo. Informed by data from Statistics Norway, we assumed the average inland transport distance in Norway to be 180 km by road.

Table S1 summarizes the raw materials, energy and transport required to produce and distribute 1 kg infant formula powder sold in Norway.

### 2.5. Consumption of Prepared Infant Formula

#### 2.5.1. Preparation of Infant Formula Ready for Consumption

To produce 1 L ready-to-feed infant formula, 0.129 kg infant formula powder is required, in addition to 0.9 L water [41]. With a water density of 0.997 kg L^−1^, 0.13 kg infant formula powder and 0.87 kg water is required to produce 1 kg ready-to-feed infant formula.

With a daily requirement of 0.82 kg ready-to-feed infant formula [45], split between five feedings, each feeding will contain 0.16 kg infant formula. We assumed that five bottles are used and sterilized daily, and that sterilizing five feeding bottles requires boiling 5 L of water for 5 min. Consequently, 1 kg ready-to-feed infant formula will provide six servings and require 6 L of water for sterilization.

For bottle sterilization, water was assumed boiled in a cooking pot on an electrical stove, with an energy consumption of 0.153 kWh L^−1^ of water, in line with Oberascher et al. [46]. For consumption, we assumed that the water was boiled by using an electric kettle with a 50% lower electricity requirement compared to using a pot on an electric stove [46].

National electricity mix from Norway was used as provided in the ecoinvent database, totally dominated by hydropower (95%).

#### 2.5.2. Production of Feeding Bottles

We assumed that five feeding bottles were used daily, and that the same five bottles were used throughout the assessed feeding period of four months (121 days). Each feeding bottle was assumed to weigh 0.045 kg. With five feeding bottles divided by a total of 99 kg infant formula consumed within the four-month period, the amount of plastic needed per 1 kg ready-to-feed infant formula was estimated to be 0.0023 kg.

#### 2.5.3. Waste

Wastage of dry goods in the grocery trade was found to be 0.5% in a report by Norwegian Institute for Sustainable Research (NORSUS) [47] and adapted for this analysis. No data were available on how much of the prepared infant formula is wasted in the household. A wastage of 15% was therefore used, based on a rough estimate of average household food waste for Europe [42].

Table S2 summarizes the raw materials and energy required to prepare 1 kg ready-to-feed infant formula in a household in Oslo, Norway.

### 2.6. Breastfeeding

#### 2.6.1. Production of Breastmilk

The environmental impact of breastfeeding is in this analysis based on the environmental impact of the additional food intake required for breastmilk production in lactating mothers.

According to the Nordic Nutrition Recommendations, production of breastmilk requires 2.6 MJ per day, based on an average milk production of 0.75 kg every 24 h produced with an energy efficiency of 80% [48,49]. The additional energy required for the lactating mother can be covered by an increased intake of dietary energy and to some degree by energy mobilization from tissues. In well-nourished women, 0.7 MJ per day may come from mobilized body fat, resulting in an additional daily energy requirement from the diet of 1.9 MJ. Recalculated into the functional unit of 1 kg breastmilk, the additional dietary energy required is 2.5 MJ per 1 kg breastmilk.

#### 2.6.2. Lactating Mother’s Food Intake

The additional 2.5 MJ dietary energy needed to produce 1 kg breastmilk has been estimated as a proportion of the average daily food intake among women of reproductive age (18–49 years) in the Norkost study. The Norkost study is a recurring national dietary surveillance survey conducted in Norway. The data used in this study were sourced from Norkost 3, which included a representative sample of adults living in Norway in November 2010, [50].

The environmental impact of the average daily food intake was calculated for the included women (*n* = 568) based on environmental impact of 66 different food and beverage categories, covering all foods consumed except alcoholic beverages. It was assumed that lactating women do not consume alcohol. In Table S3, the average food intake and energy contribution of the main food categories are presented. Environmental impact data for food items were taken from an LCA database published by the Dutch National Institute for Public Health and the Environment [38].

### 2.7. Feeding with Infant Formula and Breastfeeding for Four Months

The Norwegian Directorate of Health recommends 4–6 months exclusive breastfeeding, and most infants in Norway are introduced to complementary foods from four months [9]. We have therefore chosen four months exclusive feeding with infant formula and four months exclusive breastfeeding as a realistic real-life comparison in the Norwegian setting.

The environmental impact of exclusive feeding with infant formula for four months was based on the energy requirements of formula-fed infants, on average 2288 kJ per day during the first four months of life [45]. Assuming a daily intake of 0.82 kg infant formula, 99 kg infant formula is consumed over the whole four-month period (121 days).

The environmental impact of exclusive breastfeeding for four months was based on the energy requirements of breastfed infants, on average 2140 kJ per day during the first four months of life [45]. Assuming a daily intake of 0.76 kg breastmilk, a total of 92 kg breastmilk is consumed over the whole four-month period (121 days). We assumed breastfeeding directly at the breast and no use of breast pump or feeding bottles.

### 2.8. Sensitivity Analysis

The main ingredient in infant formula is cow milk. There is a wide variation in the environmental impact of cow milk documented in the literature, which might be related to the system boundaries applied, the allocation method or other aspects of the LCA methodology, or the actual conditions at the farm [51]. To account for variations in the environmental impact of cow milk, we have performed sensitivity analyses of the impact from cow milk, namely estimated the overall environmental impact from production of infant formula if the impact from cow milk were 25% or 50% lower than our base case scenario based on values from the Agri-footprint 5.0 database. This large variation in impacts was due to findings in the literature that compare different production strategies [51] and production in different countries [52].

The environmental impact of breastfeeding is directly linked to the lactating mother’s diet. In addition to the estimate based on current average diets, we have included four different dietary scenarios for the additional 2.5 MJ dietary energy required for production of 1 kg breastmilk, namely bread only, mixed plant-based food, mixed animal-source food, and meat only, see Table S4 for more detailed description of the scenarios.

## 3. Results

### 3.1. Environmental Impact from Infant Formula and Breastmilk

**Figure 2:**
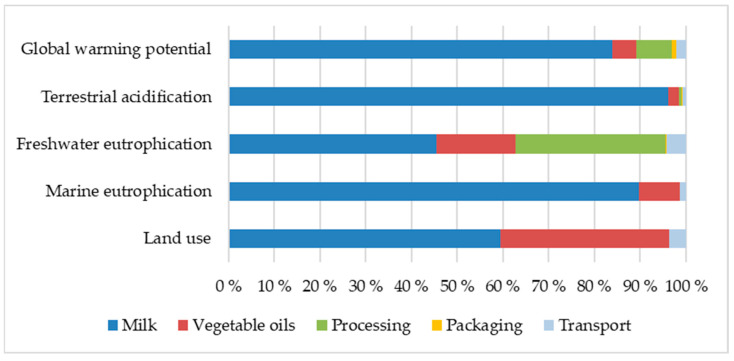
Percentage contribution to environmental impacts from production and distribution of 1 kg infant formula powder.

The estimated environmental impact of production and distribution of 1 kg milk-based infant formula intended for infants 0–6 months is presented in Table 2 below. Figure 2 shows that from production of infant formula up to retail in Norway, the production of the raw ingredient cow milk contributed most to all impact categories assessed. Its inclusion accounted for more than 80% of the global warming potential, terrestrial acidification, and marine eutrophication values. Production of vegetable oils contributed particularly to the land use value. Processing, including the energy needed for the various processes, contributed with 37% and 17% of the values for freshwater eutrophication and global warming potential, respectively, but only insignificantly to the other impact categories. Packaging and transport were found to provide minor contributions to all environmental impacts assessed in this study.

**Figure 3:**
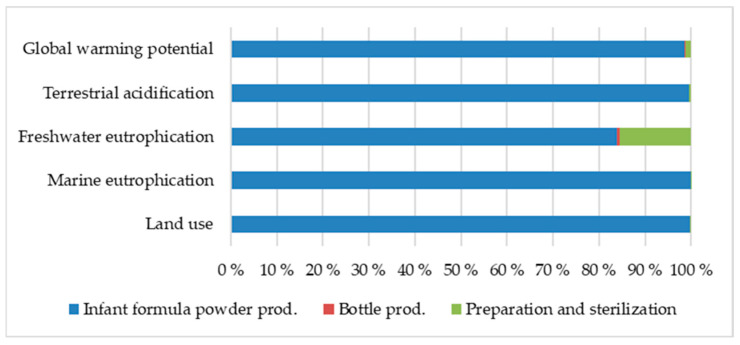
Percentage contribution to environmental impacts from 1 kg prepared infant formula ready for consumption.

The estimated environmental impact from 1 kg ready-to-feed infant formula is presented in Table 3. As shown in Figure 3, the production of the infant formula powder was the main contributor to all impact categories, constituting nearly all of the impacts on global warming potential, terrestrial acidification, marine eutrophication, and land use. Preparation of infant formula for consumption and sterilization of drinking bottles accounted for 15% of the impact of freshwater eutrophication due to the electricity needed for these processes.

The estimated environmental impact from 1 kg breastmilk is presented in Table 3 below.

### 3.2. Environmental Impact from Feeding with Infant Formula Compared to Breastfeeding

When comparing infant formula to breastmilk, the environmental impact of 1 kg ready-to-feed infant formula had a higher impact for all categories compared with 1 kg breastmilk. The differences ranged from a 24–60% higher impact of infant formula compared to breastmilk (Table 3).

After aggregating the findings presented in Table 3 to four months exclusive feeding with infant formula compared with four months exclusive breastfeeding, the difference between the two feeding methods was even more marked (Table 4). Exclusively feeding with infant formula the first four months of life resulted in a 35–72% higher score for the five impact categories than exclusively breastfeeding during the same period.

### 3.3. Sensitivity Analysis

**Figure 4:**
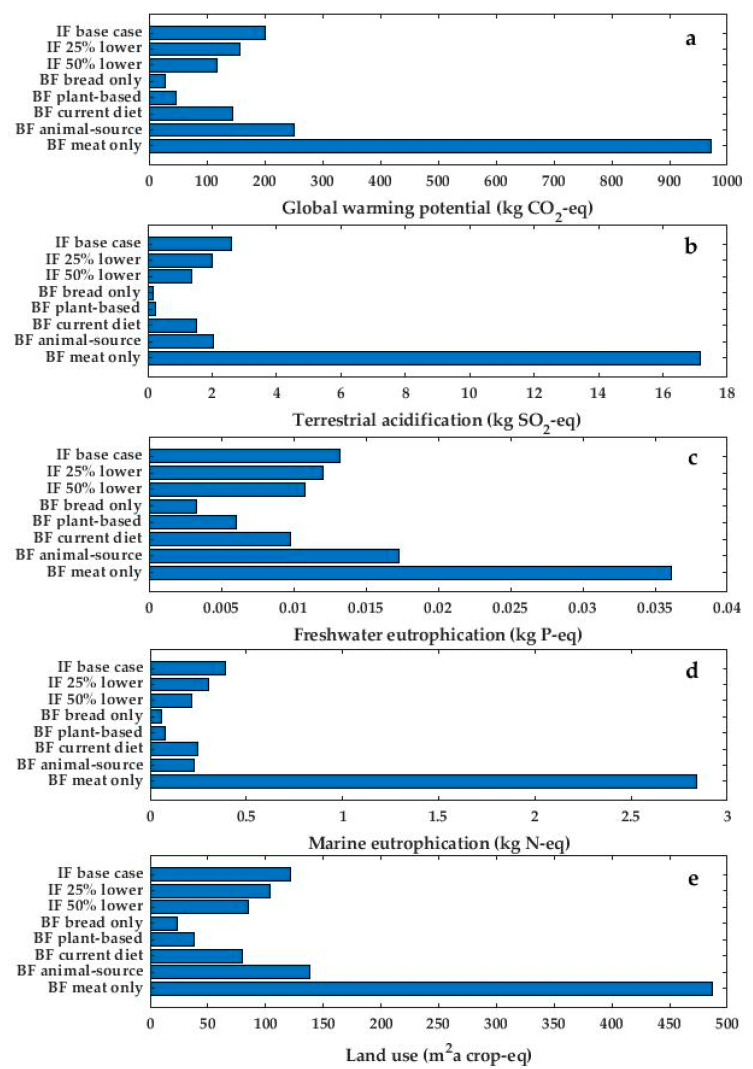
Impact from (**a**) global warming potential, (**b**) terrestrial acidification, (**c**) freshwater eutrophication, (**d**) marine eutrophication, and (**e**) land use, from four months feeding of infant formula base case and with 25% and 50% lower impact from cow milk compared to different dietary scenarios for breastfeeding. IF = infant formula; BF = breastfeeding; IF 25% lower refers to infant formula produced with 25% lower impact from cow milk; and IF 50% refers to infant formula produced with 50% lower impact from cow milk.

Results from the sensitivity analysis for both infant formula feeding and breastfeeding is presented in Figure 4a–e. Choice of a source with lower reported LCA value for cow milk considerably reduced the environmental impact from production and consumption of infant formula for all impact categories. More details are presented in Table S5.

The environmental impact of breastfeeding was directly linked to the lactating mother’s diet. For all included impact categories, the scenario with meat only gave the highest impact scores, while the scenario with bread only gave the lowest impact scores. More details are presented in Table S6.

## 4. Discussion

In the present study, we observed that for the five environmental impact categories assessed, scores were 24–60% higher for 1 kg ready-to-feed infant formula compared to 1 kg breastmilk. In addition, we found that four months feeding with infant formula compared to breastfeeding resulted in 38% higher global warming potential, 72% higher terrestrial acidification, 35% higher freshwater eutrophication, 59% higher marine eutrophication, and 53% higher land use.

Our findings are in line with Karlsson et al., 2019, who found that the global warming potential of feeding with infant formula was almost double that of breastfeeding [27]. However, these results are in contrast with those of Amonkar et al., 2019, who found instead that the global warming potential from feeding with infant formula was lower than from breastfeeding [28]. The disagreement in results can mainly be ascribed to difference in assumptions regarding how breastmilk was fed to the child. In our study and in Karlsson et al., 2019 [27], it was assumed that all breastfeeding was directly at the breast. Amonkar et al., 2019 [28], on the other hand, presumed that the lactating mother regularly expresses her breastmilk, and hence have included in their calculations production and use of electric breast pumps and feeding bottles as well as cold storing and heating of expressed breastmilk. These additional processes contributed with 18% of the total global warming potential for feeding breastmilk [28].

When assessing the sources of environmental impact from production of infant formula, production of cow milk was the single process contributing with the highest environmental score for all impact categories assessed. The contribution varied between 45% and 95% of the total score depending on the environmental impact category. The impact of cow milk varies according to production strategies at the farm, and there are large reported variations between countries and regions [52], as well as between farming practices [51]. As shown in the sensitivity analysis, a lower impact score for production of cow milk considerably reduced the overall impact of infant formula production.

In our study, we have not included the impact contribution of production of micronutrients included in the infant formula. Karlsson et al., 2019 [27] estimated that the global warming potential of the added micronutrients could be between 0.006 and 1.3 kg CO_2_-eq per kg infant formula powder, which in our case would add anywhere from 0.05% up to 10% to the global warming potential for production of infant formula powder.

The energy used during production of infant formula contributed especially to freshwater eutrophication and global warming potential. As we assumed production in the Netherlands, the impact value used in our analysis was Dutch energy mix retrieved from ecoinvent. Compared to Norwegian energy mix reported in ecoinvent, which is mainly renewable, the Dutch energy mix has more than 80% higher impact value for all impact categories, and close to double the impact score for freshwater eutrophication and global warming potential. Choosing renewable energy during processing could potentially reduce the environmental impact of processing, and the overall impact of production. As shown here, both packaging and transport have little overall impact; this is in line with values for packaging and transport of most food products [32,53].

The impact of breastfeeding was directly dependent on the lactating mother’s diet, and increased with increasing proportion of food products with animal origin. Eating plant-based foods generally results in a lower environmental impact than eating a diet dominated by animal-source foods [19,32]. If the additional dietary energy required for breastfeeding was from bread only, the impact could be around a fifth of our estimated scenario based on current average diets, whereas if the energy was solely from beef, the impact could be up to 11 times higher.

The lactating mother can therefore, by choosing a more plant-based diet, contribute to lower impact from breastfeeding for all impact categories assessed. Furthermore, the additional energy required during lactation was based on estimated average needs. In reality, a lactating mother might need more or less than this to sustain breastfeeding. If a lactating mother eats less than the estimated additional 1.9 MJ per day, the environmental impact of breastfeeding will be lower than our estimated scenario.

In the sensitivity analysis, we have illustrated that if the additional dietary energy required for breastfeeding was from beef or other animal-source food the environmental impact of breastfeeding would be higher than that of our base case for feeding with infant formula.

In the present study, we have assumed that infants fed with infant formula consume more milk per day than breastfed infants based on estimated higher energy needs of children fed infant formula [45]. Observational studies describing milk intake during the first months of life have also documented a higher consumption among infants fed formula than breastfed infants [1]. Preparing and consuming food, including infant formula, results in some degree of food waste. Parents who feed their children infant formula from a bottle are encouraged to allow for the child to regulate consumption and discard any leftover milk [54,55]. Breastfeeding directly at the breast will not leave such additional discarded milk. We have therefore included waste during both preparation and consumption of infant formula, whereas during breastfeeding we have not included any direct waste.

We have assumed that during the first four months post-partum a mother that is exclusively breastfeeding will do so by feeding directly at the breast only. Norway has a long history of providing maternity leave, and most mothers in Norway are on maternity leave the first four months post-partum [56]. We have therefore assumed that feeding directly at the breast is more common than expressing milk regularly. However, we recognize that in reality, some breastfeeding mothers choose to express milk using a breast pump regularly or occasionally in addition to feeding directly at the breast, which might raise the environmental impact of expressed breastmilk [28,57].

A strength of our study is the inclusion of five different environmental impact categories. We have found that all assessed impact categories show a similar trend of higher impact from feeding with infant formula compared to breastfeeding, as previously found by others for global warming potential. For terrestrial acidification, marine eutrophication and land use the disparities between the feeding modalities, in favour of breastfeeding, were even larger than for global warming potential. Inclusion of more impact categories also confirms that cow milk is the single most important contributor of impact in the infant formula production.

A limitation of our study is the use of secondary LCA data for the ingredients of the infant formula. A number of variables impact the LCA data for cow milk. In addition to variable conditions and practices at the farm, different system boundaries may be applied during LCA analysis, and different values for the inputs may be used. An important consideration when performing LCA on a food product is allocation. The cow milk is not the only product resulting from a cow; other products include meat from the calves produced and the cow itself. The degree of environmental impact allocated to the milk could therefore vary widely depending on the allocation. The impact scores for cow milk used in our study were retrieved from Agri-footprint and represent an average Dutch dairy farm. In comparison, the impacts used in the study by Karlsson et al., 2019 [27], from Hagemann et al., 2011, estimate as much as a 50% lower global warming potential for milk produced at Dutch dairy farms than in the Agri-footprint database [52]. As shown in the sensitivity analyses, if using an LCA data source with a lower environmental impact value for cow milk, the calculated overall environmental impact of infant formula would indeed be lower. A 50% reduction in the impact of cow milk would result in lower global warming potential, terrestrial acidification, and marine eutrophication scores for infant formula feeding than for breastfeeding.

For lactating mothers, our findings are representative for direct feeding at the breast, including impact from the mother’s diet, but not considering breast pumps or special lactation clothes. Further, as there is presently no complete LCA database for Norwegian food products, we have based the calculations on food LCA data from the Netherlands. More than 50% of food eaten in Norway is imported mainly from other European countries including the Netherlands; however, differing production conditions between internationally and domestically grown and produced food might affect the environmental impact of the lactating mother’s diet.

## 5. Conclusions

In this study, we have illustrated that four months exclusive breastfeeding directly at the breast has a lower environmental impact than four months exclusive feeding with infant formula. We observed 35–72% lower impact of breastfeeding than feeding with infant formula for the five impact categories included. The analysis indicated that cow milk was the main source of environmental impact from production of infant formula, and that the total impact would be considerably lower given a lower impact from cow milk. The environmental impact of breastfeeding was highly dependent on the composition of the lactating mother’s diet; if the additional energy was from animal-source food the impact could be more than three-five times higher than if the energy were from plant-based food only.

## Figures and Tables

**Table 1:** Assumed baseline recipe used to produce powdered infant formula, based on Karlsson et al. (2019) [27].

Ingredients	% of Solid Mass
Skimmed milk	15
Whey protein concentrate	10
Lactose	50
Vegetable oils	25

**Table 2:** Environmental impact from production and distribution of 1 kg infant formula powder.

Impact Category	Unit	1 kg Infant Formula Powder
Global warming potential	kg CO_2_-eq	12.65
Terrestrial acidification	kg SO_2_-eq	1.17 × 10^−1^
Freshwater eutrophication	kg P-eq	7.14 × 10^−4^
Marine eutrophication	kg N-eq	2.53 × 10^−2^
Land use	m^2^a crop-eq	7.82

**Table 3:** Environmental impact from 1 kg ready-to-feed infant formula and 1 kg breastmilk, and the difference between the two.

Impact Category	Unit	1 kg Infant Formula	1 kg Breastmilk	Difference between Breastmilk and Infant Formula *
Global warming potential	kg CO_2_-eq	2.02	1.58	0.44 (28%)
Terrestrial acidification	kg SO_2_-eq	2.64 × 10^−2^	1.65 × 10^−2^	0.99 × 10^−2^ (60%)
Freshwater eutrophication	kg P-eq	1.33 × 10^−4^	1.07 × 10^−4^	0.26 × 10^−4^ (24%)
Marine eutrophication	kg N-eq	3.97 × 10^−3^	2.68 × 10^−3^	1.29 × 10^−3^ (48%)
Land use	m^2^a crop-eq	1.23	0.87	0.36 (41%)

* The percentage is the difference as a proportion of breastmilk.

**Table 4:** Environmental impact from four months exclusive feeding with infant formula and four months exclusive breastfeeding, and the difference between the two.

Impact Category	Unit	4 Months Feeding with Infant Formula	4 Months Breastfeeding	Difference between Breastfeeding and Infant Formula Feeding *
Global warming potential	kg CO_2_-eq	200	145	55 (38%)
Terrestrial acidification	kg SO_2_-eq	2.61	1.52	1.09 (72%)
Freshwater eutrophication	kg P-eq	1.32 × 10^−2^	0.98 × 10^−2^	0.34 × 10^−2^ (35%)
Marine eutrophication	kg N-eq	3.93 × 10^−1^	2.47 × 10^−1^	1.46 × 10^−1^ (59%)
Land use	m^2^a crop-eq	122	80	42 (53%)

* The percentage is the difference as a proportion of breastfeeding.

## Data Availability

Dietary data for women of reproductive age was obtained from the Norkost 3 study, https://www.helsedirektoratet.no/tema/kosthold-og-ernaering/statistikk-og-undersokelser-om-ernaering (accessed on 11 August 2021). The data presented in this study are available on request from the corresponding author. LCA values for food products was obtained from a publicly available LCA database published by the Dutch National Institute for Public Health and the Environment (RIVM). This data can be found here: https://www.rivm.nl/voedsel-en-voeding/duurzaam-voedsel/database-milieubelasting-voedingsmiddelen (accessed on 29 March 2021).

## Supplementary Materials

The following supporting information can be downloaded at: https://www.mdpi.com/article/10.3390/ijerph19116397/s1, Table S1: Summary of the raw materials, energy and transport required to produce and distribute 1 kg infant formula powder; Table S2: Summary of the materials and energy required to prepare 1 kg ready-to-feed infant formula in a household in Oslo; Table S3: Average food intake in grams per day among women of reproductive age in the Norkost 3 study, and energy contribution from different food and beverage categories; Table S4: Four different dietary scenarios for the additional 2.5 MJ energy required for breastfeeding 1 kg breastmilk; Table S5: Environmental impact from four months exclusive feeding with infant formula, estimated with 25% and 50% lower impact from cow milk, compared to our base case scenario. Percentages in brackets are percentages of the base case scenario; Table S6: Environmental impact from four months exclusive breastfeeding based on different diet scenarios for the lactating mother.
