# Supplementary material for: Environmental Impact of Feeding with Infant Formula in Comparison with Breastfeeding
Source: Int J Environ Res Public Health. 2022 May 24;19(11):6397. doi: 10.3390/ijerph19116397 (PMC9180168; doi:10.3390/ijerph19116397)
Supplement: Supplementary file 1 [file ijerph-19-06397-s001.zip › ijerph-1691456-supplementary.pdf]

International Journal of
Environmental Research and Public Health

MDPI

Supplementary Material – Environmental Impact of Feeding with Infant Formula in Comparison with Breastfeeding

Table S1. Summary of the raw materials, energy and transport required to produce and distribute 1 kg infant formula powder.

Input	Unit	1 kg powdered infant formula	Source/Description
Raw milk	kg ECM[1]	6.57	Karlsson et al. (2019)
Sunflower oil	kg	0.21	Tietze (2021)
Rapeseed oil	kg	0.04	Tietze (2021)
Electricity	kWh	0.69	Karlsson et al. (2019)
Natural gas	MJ	7.05	Karlsson et al. (2019)
Canning steel	kg	0.14	Our estimate
Sea transport	tkm[2]	1.45	Our estimate
Train transport	tkm	0.04	Our estimate
Road transport	tkm	0.45	Our estimate
Refrigerated road transport	tkm	0.67	Our estimate

[1] ECM = energy corrected milk
[2] tkm = tonne-kilometre, which is the transport of 1 tonne of product over a distance of 1 km

Table S2. Summary of the materials and energy required to prepare 1 kg ready-to-feed infant formula in a household in Oslo.

Ingredient/Energy	Unit	Amount	Amount including waste	Source/Description
Infant formula powder	kg	0.13	0.16	Nestlé website[1]
Feeding bottles (HDPE)	kg	0.0023	0.0023	Our estimate
Electricity for sterilization	kWh	0.92	0.92	Our estimate, based on Oberascher et al. (2011)
Electricity for preparation	kWh	0.07	0.08	Our estimate, based on Oberascher et al. (2011)

[1] www.nestlebarnemat.no/produkt/nan-pro-1-800g

Table S3. Average food intake in grams per day among women of reproductive age in the Norkost 3 study, and energy contribution from different food and beverage categories (Myhre et al. 2020).

Food item	Food intake, g per day	Energy contribution, %
Total	3643	100 (8239 kJ)
Bread	151	20
Pasta, rice and cereals	41	7
Cakes	33	6
Potatoes	46	2
Vegetables	155	3
Fruit and berries	170	7
Fruit juice	114	3
Meat and meat products	120	11
Pork	49	5
Beef	35	3
Poultry	29	2
Lamb	7	1
Fish and fish products	48	4
Fatty fish	24	2
Lean fish	20	1
Shellfish	3	0
Egg	21	1
Milk and dairy products	276	9
Milk	223	5
Yoghurt	32	2
Cream	21	2
Cheese	45	7
Butter and margarine	24	7
Soya milk	6	0
Sugar and sweets	20	4
Coffee	401	0
Tea	226	0
Lemonade and soda	246	2
Bottled water	75	0
Tap water	1134	0
Alcoholic beverages	86	2
Snacks	6	1
Other [1]	104	2

[1] Other includes almost 90% water in meals, in addition to sauces, gravy, herbs and spices.

Table S4. Four different dietary scenarios for the additional 2.5 MJ energy required for breastfeeding 1 kg breastmilk.

Scenarios	Food items
Scenario 1: Bread only	350 g bread
Scenario 2: Mixed plant-based food	160 g bread, 18 g margarine, 330 g fruit, 460 g vegetables
Scenario 3: Mixed animal-source food	330 g milk, 80 g egg, 130 g chicken, 130 g salmon
Scenario 4: Meat only	210 g bovine meat steak, 210 g bovine meat minced

Table S5. Environmental impact from four months exclusive feeding with infant formula, estimated with 25% and 50% lower impact from cow milk, compared to our base case scenario. Percentages in brackets are percentages of the base case scenario.

Impact category	Unit	Base case scenario	25% lower impact from cow milk	50% lower impact from cow milk
Global warming potential	kg CO_2-eq	200	158 (79%)	117 (59%)
Terrestrial acidification	kg SO_2-eq	2.61	1.99 (76%)	1.37 (52%)
Freshwater eutrophication	kg P-eq	1.32×10^{-2}	1.20×10^{-2} (91%)	1.07×10^{-2} (81%)
Marine eutrophication	kg N-eq	3.93×10^{-1}	3.05×10^{-1} (78%)	2.17×10^{-1} (55%)
Land use	m^2a crop-eq	122	104 (85%)	85 (70%)

Table S6. Environmental impact from four months exclusive breastfeeding based on different diet scenarios for the lactating mother.

Impact category	Unit	Bread only	Plant-based mixed	Current average diet	Animal-source mixed	Meat only
Global warming potential	kg CO_2-eq	28	46	145	252	972
Terrestrial acidification	kg SO_2-eq	0.16	0.26	1.52	2.04	17.14
Freshwater eutrophication	kg P-eq	0.33×10^{-2}	0.60×10^{-2}	0.98×10^{-2}	1.73×10^{-2}	3.61×10^{-2}
Marine eutrophication	kg N-eq	0.06	0.08	0.25	0.23	2.84
Land use	m^2a crop-eq	24	38	80	138	487

References

1. Karlsson, J.O.; Garnett, T.; Rollins, N.C.; Roos, E. The carbon footprint of breastmilk substitutes in comparison with breastfeeding. J Clean Prod 2019, 222, 436-445, doi:10.1016/j.jclepro.2019.03.043.
2. Myhre, J.B.; Andersen, L.F.; Kristiansen, A.L. Landsomfattende undersøkelse av kostholdet blant spedbarn i Norge, 6 måneder; Folkehelseinstituttet: Oslo, 2020.
3. Oberascher, C.; Stamminger, R.; Pakula, C. Energy efficiency in daily food preparation. Int J Consum Stud 2011, 35, 201-211, doi:10.1111/j.1470-6431.2010.00963.x.
4. Tietze, L. (Nestlé Danmark, Copenhagen, Denmark). Personal communication, 9 April 2021.
